# The Mitochondrial Genome of the Leaf-Cutter Ant *Atta laevigata*: A Mitogenome with a Large Number of Intergenic Spacers

**DOI:** 10.1371/journal.pone.0097117

**Published:** 2014-05-14

**Authors:** Cynara de Melo Rodovalho, Mariana Lúcio Lyra, Milene Ferro, Maurício Bacci

**Affiliations:** 1 Centro de Estudos de Insetos Sociais, UNESP – Univ Estadual Paulista. Rio Claro, São Paulo, Brazil; 2 Instituto Oswaldo Cruz, FIOCRUZ – Fundação Oswaldo Cruz. Rio de Janeiro, Rio de Janeiro, Brazil; 3 Departamento de Zoologia, UNESP – Univ Estadual Paulista. Rio Claro, São Paulo, Brazil; 4 Departamento de Bioquímica e Microbiologia, UNESP – Univ Estadual Paulista. Rio Claro, São Paulo, Brazil; BiK-F Biodiversity and Climate Research Center, Germany

## Abstract

In this paper we describe the nearly complete mitochondrial genome of the leaf-cutter ant *Atta laevigata*, assembled using transcriptomic libraries from Sanger and Illumina next generation sequencing (NGS), and PCR products. This mitogenome was found to be very large (18,729 bp), given the presence of 30 non-coding intergenic spacers (IGS) spanning 3,808 bp. A portion of the putative control region remained unsequenced. The gene content and organization correspond to that inferred for the ancestral pancrustacea, except for two tRNA gene rearrangements that have been described previously in other ants. The IGS were highly variable in length and dispersed through the mitogenome. This pattern was also found for the other hymenopterans in particular for the monophyletic Apocrita. These spacers with unknown function may be valuable for characterizing genome evolution and distinguishing closely related species and individuals. NGS provided better coverage than Sanger sequencing, especially for tRNA and ribosomal subunit genes, thus facilitating efforts to fill in sequence gaps. The results obtained showed that data from transcriptomic libraries contain valuable information for assembling mitogenomes. The present data also provide a source of molecular markers that will be very important for improving our understanding of genomic evolutionary processes and phylogenetic relationships among hymenopterans.

## Introduction

*Atta laevigata* Smith, 1858 (Hymenoptera: Formicidae: Attini) is a crop pest that is found throughout South America and is widely distributed in Brazil [1], [2]. The prevalence of this agricultural pest is related to its high population density [3] and long life span of the queens [4], resulting in the requirement for a large amount of fresh plant material to maintain the nest. The species cuts leaves from both monocotyledons and dicotyledons plants, including many plantations [5]–[7], as well as a wide variety of native plants from different biomes such as the Cerrado or the rainforest [8], [9]. It is easily recognized based on the very large, shiny head of the soldiers, a characteristic that has led to the popular name “cabeça de vidro” (meaning glass head) in Brazil.

In an aim to better understand the molecular bases of *A. laevigata* biology, physiology, behavior, and social life, and to find more specific strategies to control the pest, we recently published a partial transcriptome of this species using Sanger sequencing technology [10]. A more complete transcriptome using the Illumina platform is currently being annotated (unpublished data). Characterization of the transcriptome resulted in the retrieval of a large number of mitochondrial sequences. Although ants are highly diverse and represent an ecologically dominant group in terrestrial ecosystems [11], mitogenomes have been described and annotated for only *Pristomyrmex punctatus* [12] and three species of *Solenopsis* [13]. The mitogenome of *Atta cephalotes* [14] is available in GenBank (HQ415764) but annotation is missing, and the mitochondrial genome of *Camponotus chromaiodes* is not complete in GenBank (JX966368).

Animal mitochondrial DNA (mtDNA) has been used extensively to investigate population structures and in evolutionary and phylogenetic studies at various taxonomic levels, validating its utility as a molecular marker for systematics [15]–[17]. A growing interest in the reconstruction of phylogenetic relationships in Hymenoptera using mitochondrial genomes together with technological improvements and reduced DNA sequencing costs has led to a rapid increase in the number of sequenced mitogenomes [18]–[20].

For many years, mitogenomes were obtained by isolating mitochondria followed by DNA extraction, a procedure that is effective for large organisms but not for small organisms and some tissues [21]. To overcome this and other obstacles, long-range PCR combined with primer walking sequencing has become an alternative approach [21], [22]. More recently, next-generation sequencing (NGS) has been used to generate mtDNA data [20], [21], [23], [24], and expressed sequence tags have been useful for annotating and validating mitochondrial genomes [25].

Here, we describe the mitochondrial genome of a species from the Attini tribe, the leaf-cutter ant *A. laevigata*, using sequences obtained from transcriptomic libraries followed by PCR procedure to fill in sequence gaps and confirm intergenic regions.

## Methods and Materials

### Obtaining mitochondrial sequences from transcriptomic libraries

We retrieved mitochondrial sequences from two transcriptomic libraries of *A. laevigata*, each generated using a pool of soldiers from a single monogynic nest: a Sanger sequencing library (SL) [10] from ants collected in Rio Claro, SP, Brazil (W 22°23.716' and S 47°32.533'); and an Illumina platform library (IL) from ants collected in Botucatu, SP, Brazil (W 48°26.156′ and S 22°50.250′). Despite the fact the ants were collected in different locations, they belong to the same regional group (unpublished data), which is different from those groups previously described [26] based on mitochondrial haplotypes. The ants were collect with IBAMA permit SISBIO 33487-2 and do not involve endangered or protected species and protected area.

The SL data were pre-processed and assembled using the automated pipeline generation system EGene [27]. Sequences of vector (pDONR222) and primer (M13F) were trimmed and high quality sequences (base quality with phred ≥ 20) were selected and assembled into contigs and singlets using the CAP3 software [28], with an *overlap percent identity cutoff* “p” of 90 and a *minimum overlap length cutoff* “o” of 50. Functional annotation was based on BLASTX search of contig nucleotide sequences against the non-redundant protein database (nr) of NCBI, performed under the default settings of BLAST2GO [29] and the BLAST E-value of 1.0e^−5^ and maximum of 20 hits.

For IL, total RNA was extracted using Trizol protocol (Invitrogen). The library was constructed and sequenced at Fasteris SA, in Swiss. The total RNA quality, concentration, and integrity were determined using Qubit Analyzer (Invitrogen) and Bioanalyzer (Agilent). The paired-end library was sequenced in HiSeq 2000 in a single lane of 50 base reads. IL data were submitted to *de novo* assembly using VELVET [30] with the parameter kmer 43 and the contigs were filtered using BLAST search against ant mitochondrial genes.

For both libraries, contigs were manually verified to exclusion of homopolymer regions to avoid error in the inference of the genomic sequence. All mitochondrial sequences were then mapped onto the mitogenomes of Hymenoptera to generate a first draft of *A. laevigata* mitogenome (i.e., a mitogenome with gaps), which was used to design new primers for protein coding genes completion and amplification of intergenic regions (described below).

**Figure 1:**
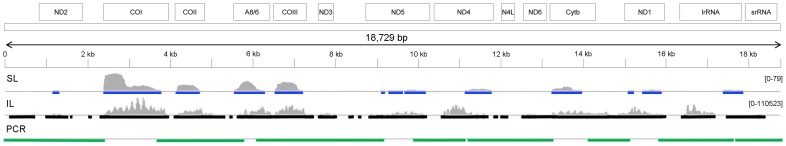
Contribution of transcriptomic libraries and PCR technique for the assembling of *A. laevigata* mitochondrial genome. The figure displays the relative position of the protein coding-genes and ribosomal subunits and the contribution of Sanger library (SL – in blue), Illumina library (IL - black), and PCR fragments (PCR - green) for the final mitogenome assembling. The grey picks represent number of sequences for each codon position in different scale (values between square brackets). The figure is an adaptation of the files generated by Bowtie2 and SAMTools and visualized using IGV program.

All sequences obtained by transcriptomic libraries and PCR were mapped into the final mitogenome sequence to access the relative cover of each technique (SL, IL, and PCR; Figure 1). For this, we used Bowtie2 [31] and SAMTools [32] and the results were visualized using IGV version 2.3.18 [33].

### Filling the gaps: amplifying and sequencing intergenic regions

Universal and new primers used to fill in the mitochondrial sequence gaps are shown in Table S1 and Figure S1. New primers were designed based on the obtained SL and IL sequences and mapped onto the Hymenoptera mitogenomes. Template DNA was extracted from a single soldier from the Botucatu nest (see below) according to Martins et al. [34]. The PureTaq Ready To Go kit (GE Healthcare) was used for PCR reactions, in total volume of 25 µL, containing 5 pmol of each primer, and ∼100 ng of template and included an initial denaturation of 3 min at 94°C followed by 35 cycles of 30 s at 94°C, 30 s at 45–58°C, and 90 s at 60°C. Amplicons were visualized in a 1% agarose gel, purified using GFX PCR DNA and Gel Band Purification Kit (GE Healthcare), quantified using a NanoDrop 2000 (Thermo Scientific), and sequenced. Amplicons that could not be directly sequenced were cloned into *Escherichia coli* DH10B using the CloneJET PCR Cloning Kit (Fermentas), and the clones were sequenced. Bidirectional sequences were generated with ABI 3500 (Applied Biosystems), trimmed with EGene system [27], and filtered by length (>100 bp) and quality (phred >20 and 90% minimum identity of window).

All intergenic regions, as well as tRNA and rRNA were obtained or confirmed by sequenced PCR products.

### Genome assembly, annotation and analysis

Final mitogenome assembly was based only on IL sequences and PCR fragments obtained from individuals from Botucatu to avoid population polymorphisms. IL and PCR data were aligned using CAP3 [28] and annotated with the program DOGMA [35] and the web server MITOS [36]. The coding regions and ribosomal subunits were manually verified by comparison with two ant mitochondrial genomes (*Solenopsis invicta*, NC_014672 and *Pristomyrmex punctatus*, NC_015075) using MEGA version 5 [37]. The sequence data for all coding genes were translated into amino acids to confirm the absence of premature stop codons, i.e., to preclude the sequencing of nuclear mtDNA pseudogenes (numts). Validation of tRNA sequences was performed using the programs tRNAScan-SE [38] and ARWEN [39]. Codon usage, aminoacid translation, A+T content, and base composition for each codon position were obtained using MEGA version 5 [37].

### Phylogenetic analysis and comparison of intergenic spacers

We used a Bayesian analysis, as implemented in BEAST software v1.7.5 [40], to infer species relationships following Mao et al. [20]. Mitogenomic sequences for 24 hymenopteran species and two non-hymenopteran were obtained from GenBank (Table 1). Only hymenopteran mitogenomes that were complete for protein-coding and rRNA genes were included in the analyses (24 out of 36 available in Genbank in September 20, 2013).

**Table 1:** Taxonomy, GenBank accession numbers, and mitogenome sizes of Hymenoptera mitochondrial genomes used for the phylogenetic analysis.

Order	Family	Species	GenBank N°	Genome size (bp)	IGS bp (N)*	Reference
Diptera	Calliphoridae	*Cochliomyia hominivorax*	NC_002660	16,022	120 (14)	[41]
Lepidoptera	Bombycidae	*Bombyx mandarina*	NC_003395	15,928	361 (13)	[42]
Hymenoptera						
Symphyta	Cephidae	*Cephus cinctus*	NC_012688	19,339	311 (20)	[19]
	Orussidae	*Orussus occidentalis*	NC_012689	15,947	127 (12)	[19]
	Tenthredinidae	*Monocellicampa pruni*	JX566509	15,169	427 (18)	[43]
Apocrita	Apidae	*Apis cerana*	NC_014295	15,895	767 (23)	[44]
	Apidae	*Apis florea*	NC_021401	17,694	939 (28)	[45]
	Apidae	*Apis mellifera ligustica*	NC_001566	16,343	813 (24)	[46]
	Apidae	*Bombus hypocrita sapporensis*	NC_011923	15,468	1,214 (21)	[47]
	Apidae	*Bombus ignitus*	NC_010967	16,434	1,063 (24)	[48]
	Apidae	*Melipona bicolor*	NC_004529	14,422	477 (16)	[49]
	Braconidae	*Cotesia vestalis*	NC_014272	15,543	252 (24)	[50]
	Braconidae	*Spathius agrili*	NC_014278	15,425	155 (15)	[50]
	Crabronidae	*Philanthus triangulum*	NC_017007	16,029	217 (11)	[51]
	Evaniidae	*Evania appendigaster*	NC_013238	17,817	948 (15)	[52]
	Formicidae	*Pristomyrmex punctatus*	NC_015075	16,180	779 (28)	[12]
	Formicidae	*Solenopsis geminata*	NC_014669	15,552	523 (24)	[13]
	Formicidae	*Solenopsis invicta*	NC_014672	15,549	519 (25)	[13]
	Formicidae	*Solenopsis richteri*	NC_014677	15,560	523 (25)	[13]
	Formicidae	*Atta laevigata*	KC_346251	18,729	3,808 (30)	Present study
	Ichneumonidae	*Diadegma semiclausum*	NC_012708	18,728	1,846 (13)	[53]
	Ichneumonidae	*Enicospilus sp.*	FJ478177	15,300	281 (14)	[19]
	Mutillidae	*Radoszkowskius oculata*	NC_014485	18,442	652 (13)	[53]
	Scelionidae	*Trissolcus basalis*	JN903532	15,768	276 (19)	[20]
	Vanhorniidae	*Vanhornia eucnemidarum*	NC_008323	16,574	2,626 (23)	[54]
	Vespidae	*Abispa ephippium*	NC_011520	16,953	1,428 (26)	[55]
	Vespidae	*Polistes sp.*	EU024653	14,741	660 (20)	[55]

*IGS bp: sum of intergenic spacers. N: number of intergenic regions in complete mitogenome (excluding A+T rich region).

Each protein-coding and ribosomal RNA gene was aligned in MEGA version 5 [37] using Muscle [56]. Small portions of clearly missed homologous regions were corrected manually. Data were divided into four partitions: the first, second, and third codon positions and the rRNA genes. The best-fit model GTR+I+G was chosen for all of the partitions and was estimated with MEGA version 5 using a likelihood ratio test according to the Bayesian information criterion. We performed two analyses: one using all partitions and the other excluding the third codon position. The Yule model, starting with a randomly generated tree, was used as a baseline model. The chains were run for 50 million generations, and the tree parameters were sampled every 5,000 generations; 25% of the initial values were discarded as burn-in. Convergence of the runs was confirmed using Tracer v1.4 [57], and the tree was summarized in TreeAnotator v1.6.2 [58] using the maximum clade credibility option as target tree type and mean heights for the node heights.

For all mitogenomes included in the analyses we compared size and number of all available intergenic spacers (IGS), excluding the putative control region after the *srRNA* gene.

## Results and Discussion

### Comparison between transcriptomic libraries

Sanger or Illumina libraries were good sources of mitochondrial sequences, providing 45% and 78% of the *A. laevigata* mitogenome, respectively (Table 2 and Figure 1).

**Table 2:** Comparison of the transcriptomic libraries for the assembling of *A. laevigata* mitochondrial genome.

Gene	Illumina Library		Sanger Library	
	Reads	bp*	Reads	bp
*trn VMIQ*	15,573	667	0	0
*NAD2*	19,993	555	0	0
*trn WCY*	675	164	0	0
*COI*	692,055	657–117–150–368	123	1,436
*COII*	179,406	447	30	643
*COII-trn KD*	68,731	693	0	0
*ATP8-6*	121,623	239–155	47	966
*ATP8-6-COIII*	162,863	409	0	0
*COIII*	236,772	185–114–315	43	722
*NAD3*	12,569	162	0	0
*NAD3-trn ARNSEF*	7,614	321	0	0
*trn ARNSEF*	617	159	0	0
*NAD5*	225,379	1,552	9	1,449
*NAD4*	371,624	1,302	11	826
*NAD4L*	2,603	368	0	0
*NAD6*	18,327	415	0	0
*NAD6-Cytb*	47,000	439	0	0
*Cytb*	97,794	289–108	21	970
*Cytb-trnS*	136,312	935	0	0
*NAD1*	290,019	999	6	1,365
*trnL-lrRNA*	8,217	329	0	0
*lrRNA*	292,532	861	0	0
*lrRNA-srRNA*	18,127	1,036	0	0
*srRNA*	2,955	274	0	0
**Total**	**3,029,380**	**14,784**	**290**	**8,377**

*Number of base pairs for each contig. Sizes of non-overlapping contigs for a given gene are separated by a dash.

However, the two sequencing technologies employed herein were very different with respect to sample preparation, time of work with hands on, cost and amount of data generated. SL consumes many work hours (cloning and sequencing) and yields few sequences compared with IL, which can generate millions of reads in a few days with lower costs [59], [60]. Consequently, IL provided greater coverage (14,784 bp) than SL (8,377 bp), resulting in less effort to fill in the remaining sequence gaps. In contrast, SL had the advantage of generating longer reads (average of 931 bp) than IL (average of 462 bp), which facilitated the bioinformatics assembly process. For the *COI* and *COIII* genes, IL generated many short and non-overlapping contigs, whereas SL resulted in a single large contig (Table 2). However, IL provided a better indication of gene expression because it generated hundreds or thousands of reads for each gene compared to SL (Figure 1). Table 2 shows that SL recovered 8,377 reads from eight protein-coding genes, whereas IL recovered 2.21 million reads from the same genes. In addition, IL recovered tRNA and ribosomal subunit genes with reduced expression levels that were not sampled using SL.

### Sequence composition

A single 18,729 bp sequence was obtained for the *A. laevigata* mitogenome and submitted to GenBank (KC346251). This sequence is incomplete in the AT-rich control region, which has an estimated size about 150–300 bp based on the length of amplicons. We were unable to sequence this region, which has been shown to be difficult to amplify and sequence in Hymenoptera [19], [54], [55]. We identified the same 37 genes present in other animals: 13 protein-coding genes, two rRNAs, and 22 tRNA genes (Table 3) [61], [62]. Twenty-three genes were encoded by the majority strand (J strand, [63]); 14 were encoded by the opposite (N) strand (Table 3).

**Table 3:** Mitochondrial genome annotation and A+T content of *A. laevigata*.

Gene	Position*	Size (bp)	IGS (bp)#	AT (%)	Start	Stop
*trnV*	(21–89)	69	101	88.4	-	-
*trnM*	191–261	71	166	72.5	-	-
*trnI*	428–499	72	93	82.6	-	-
*trnQ*	(593–662)	70	189	79.7	-	-
*ND2*	852–1832	981	8	87.0	ATT	TAA
*trnW*	1841–1910	70	11	85.5	-	-
*trnC*	(1922–1991)	70	118	97.1	-	-
*trnY*	(2110–2175)	66	202	84.8	-	-
*COI*	2378–3910	1,533	160	70.2	ATG	TAA
*trnL_2_*	4071–4141	71	0	78.3	-	-
*COII*	4142–4825	684	196	73.7	ATT	TAA
*trnK*	5022–5091	70	236	82.6	-	-
*trnD*	5328–5396	69	167	88.4	-	-
*ATP8*	5564–5747	184	1	84.2	ATA	T
*ATP6*	5749–6414	666	91	76.4	ATA	TAG
*COIII*	6506–7297	792	215	70.0	ATG	TAA
*trnG*	7513–7577	65	0	93.8	-	-
*ND3*	7578–7931	354	57	78.8	ATT	TAA
*trnA*	7989–8054	66	85	87.9	-	-
*trnR*	8140–8213	74	207	87.0	-	-
*trnN*	8421–8490	70	−3	82.6	-	-
*trnS_1_*	8488–8548	61	−1	83.9	-	-
*trnE*	8548–8615	68	−8	95.6	-	-
*trnF*	(8608–8676)	69	13	91.3	-	-
*ND5*	(8690–10354)	1,665	0	79.7	ATT	TAA
*trnH*	(10355–10427)	73	8	82.6	-	-
*ND4*	(10436–11782)	1,347	247	80.8	ATA	TAG
*ND4L*	(12030–12305)	276	11	86.9	ATT	TAG
*trnT*	12317–12386	70	1	89.9	-	-
*trnP*	(12388–12460)	73	84	87.0	-	-
*ND6*	12545–13105	561	70	84.0	ATG	TAA
*Cytb*	13176–14294	1,119	257	73.8	ATG	TAA
*trnS_2_*	14552–14621	70	322	87.0	-	-
*ND1*	(14944–15891)	948	176	78.6	ATA	TAA
*trnL_1_*	(16068–16138)	71	221	81.2	-	-
*lrRNA*	(16360–17785)	1,426	95	83.1	-	-
*srRNA*	(17881–18675)	795	74+	85.5	-	-
**Total**		**18,729**	**3,882**	**80.8**		

*The J strand is used as reference for position numbers. Parentheses indicate genes encoded by the N strand.

# Non-coding intergenic spacer between two adjacent genes. Negative numbers indicate the overlap size in base pairs.

+ Incomplete sequence.

The A+T content of mitogenome, missing the unsequenced region, was 80.8% (Table 3), which is higher than that found in *Solenopsis* (77%) and in *Pristomyrmex* (79.6%) and is consistent with the pattern described for Hymenoptera [55], [13]. Distinct parts of the mitogenome displayed an A+T content that varied from 70% (*COIII*) to 97.1% (*trnC*).

Protein-coding genes had an A+T content of 78.8%, which is less than that characterizing the entire genome sequence, as previously shown in *Apis mellifera* [46] and in *Solenopsis* [13]. At the third codon position, the A+T content (86.4%) was higher than that of the whole mitogenome; the A+T content of the first and second positions was lower (76.3% and 73.6%, respectively), as reported for other insects [20], [25], [54], [64].

This AT-bias was reflected by the codon usage, as the mitogenome was found to be highly skewed towards codons that are high in A+T content. The four most represented codons were ATT for isoleucine, TTA for leucine, TTT for phenylalanine and ATA for methionine, while codons rich in C and G, such as CTG for leucine, AGC for serine, CGC for arginine and TGC for cysteine, were rarely or never used.

In agreement with *Solenopsis* mtDNA [13], T-bias was high in all protein-coding regions, especially in the second codon position. There was a discrepancy between these two genomes with respect to G content, which was lower in *A. laevigata* at all positions.

The A+T content of *srRNA* and *lrRNA* was 85.5% and 83.1%, respectively (Table 3), and although we lack some information regarding the A+T content of the control region, these values are consistent with that found in other Hymenoptera that commonly display an elevated A+T content for ribosomal subunits compared with total mtDNA [54], [64]. The *srRNA* and *lrRNA* genes of *A. laevigata* (795 bp and 1,426 bp, respectively) were slightly longer than those of *S. invicta* and *P. punctatus*. The precise ends of these rRNAs were difficult to determine because they are usually defined based on the surrounding coding genes or tRNAs (see [19]). In addition, in *A. laevigata*, there were non-coding sequences surrounding both genes (IGS, see below).

### Mitogenome organization

**Figure 2:**
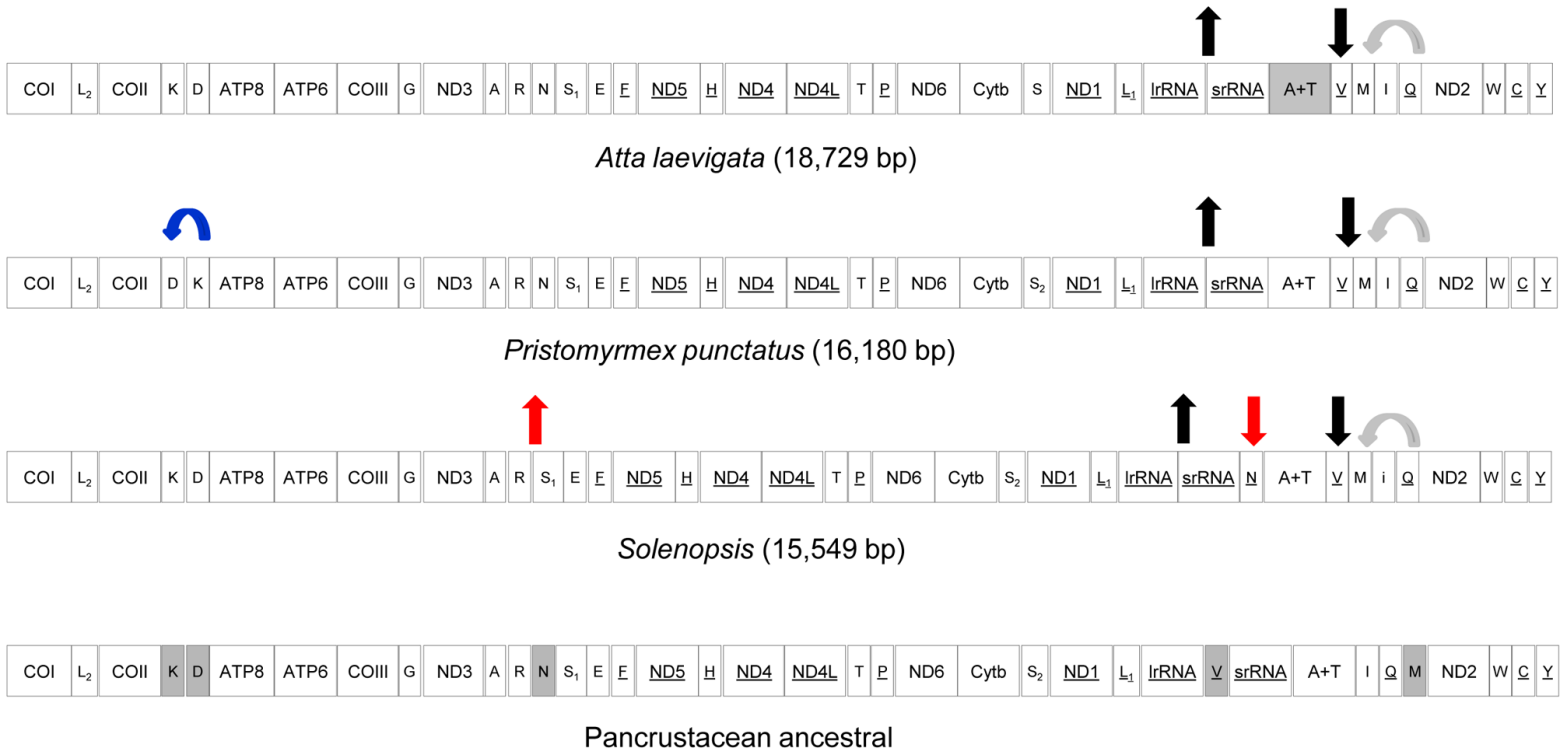
Organization of the *A. laevigata* mitogenome compared with those of the ancestor and other ants. All protein and rRNA-coding genes are in the same direction and position found in other Hymenoptera and hypothetical pancrustacean ancestral sequences. Genes encoded by the N strand are underlined; the remaining genes are encoded by the J strand. The control region of *A. laevigata* (gray) is incomplete. Shaded genes in pancrustacean ancestral sequence indicate rearrangements and arrows indicate position shifts of tRNA genes compared to it. Black arrow: *trnV* translocation from the *lrRNA-srRNA* junction to the *srRNA-ND2* junction; grey arrow: *trnI-trnQ-trnM* became *trnM-trnI-trnQ*; blue arrow: *trnK* and *trnD* swapped positions; red arrow: *trnN* translocation from the *trnA-trnR-trnN-trnS_1_-trnE-trnF* cluster to a position upstream of *srRNA*, with an inversion. This figure was adapted from Gotzek et al. [13].

Protein-coding genes and rRNA genes in *A. laevigata* displayed the same order and orientation as those present in the hypothesized ancestral pancrustacean mitogenome [16], [64], [65] (Figure 2). However, the locations of *trnV* and *trnM* indicated distinct rearrangements, as previously reported for *P. punctatus* and *Solenopsis* [12], [13]. The position occupied by *trnV* is uncommon in other Hymenoptera mitogenomes but was recently reported in the wasp *Trissolcus basalis* [20]. Although these three ants belong to Myrmicinae, *Solenopsis* and *P. punctatus* display other rearrangements that are not detected in *A. laevigata* (Figure 2). Rearrangements of tRNAs are a typical feature of the hymenopteran mitogenome architecture [19], [55].

All of the predicted tRNA molecules had the typical cloverleaf structure excluding *trnS_1_* (data not shown). In that case, the dihydrouridine arm formed a simple loop, as observed in several species including insects [54], [66]. The tRNA molecules varied between 61 (*trnS_1_*) and 74 bp (*trnR*), and the anticodons were identical to those described for *Solenopsis* [13] excluding *trnN*, which consisted of GTT rather than the ATT anticodon found in *Solenopsis*.

We found only three overlapping regions in the *A. laevigata* mtDNA (Table 2), and all of them were positioned between tRNA genes: a three-nucleotide overlap between *trnN* and *trnS_1_,* one between *trnS_1_* and *trnE*, and eight between *trnE* and *trnF* (these last two genes occupied different strands). Although it is common to see overlaps between tRNAs and protein-coding genes or between proteins and protein-coding genes (e.g., [25], [54], [64]), overlaps were detected only between tRNAs in *A. laevigata*.

The start codons ATG, ATA or ATT are common initiation sites in invertebrate mitochondrial genomes [20], [54], [64] and can be assigned to all protein-coding genes (Table 2). The majority of protein-coding genes were predicted to end in TAA, and only three genes (*ATP6, ND4, ND4L*) terminated with the stop codon TAG. *ATP8* lacks a complete stop codon and appears to terminate with a single T from which a stop codon could be created by post-transcriptional polyadenylation, as observed in other animals [67]–[70].

### Phylogenetic analyses and intergenic spacers

**Figure 3:**
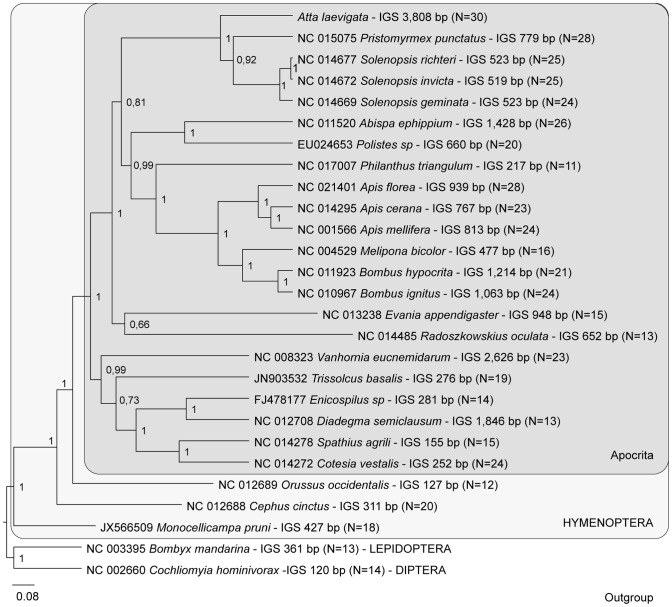
Bayesian tree derived from mitogenomic analyses. Dataset included first and second codon positions from protein-coding genes and the rRNA genes. Posterior probabilities are indicated at each node. IGS: sum of intergenic spacers in base pairs. N = number of intergenic spacers.

The tree derived from Bayesian inference analyses of the mitochondrial protein-coding gene and rRNAs is shown in Figure 3. The topologies obtained with and without third codon positions were broadly congruent. But the analysis excluding the third codon positions recovered the Apocrita as a monophyletic group, while the analysis with all codon positions recovered a controversial clade, with *Vanhornia eucnemidarum* out of the Apocrita (Figure S2). This is consistent with previous studies that suggest that the exclusion of the third codon position improves phylogenetic analyses using hymenopteran mitogenomes [71], [51], [20]. The analyses recovered most of the expected relationships on Hymenoptera (according [72]). However, the results obtained here do not support the monophyly of Aculeata (see [72]) because of the position of *Radoszkowskius aculata* (Aculeata: Mutillidae). Similar result was obtained previously by Kaltenpoth and colleagues [51], and it can be due to a long-branch attraction phenomenon [73] or the inclusion in the analysis of a small number of taxa containing complete genome data.

A remarkable feature of the *A. laevigata* mitogenome was the presence of IGS spanning 3,808 bp and comprising an average A+T content of 86.1% (Table 3). IGS occurred between almost all of the genes, i.e., in 30 out of the 37 possibilities. Fourteen of them consisted of more than 160 bp, and the longest one contained 322 bp and was located between the *trnS_2_* and *ND1* genes. The sizes of these IGS were considerably greater than those commonly found in other insect mtDNAs, which display non-coding nucleotides outside the control (AT-rich) region that are smaller than 50 bp [54].

Unique or few large non-coding intergenic sequences, which are commonly repeated sequences, have been reported to mollusks, nematodes and arthropods, causing their mitogenomes to reach sizes of up to 40 kb [61], [74], [75]. In contrast, the IGS in *A. laevigata* were relatively short, variable in length, lacked repeats, and were abundantly dispersed through the 19 kb mitogenome. This same pattern was found for the other hymenopteran mitogenomes analyzed here, in particular for the monophyletic Apocrita (Table 1, Figure 3). Despite the fact that the mitochondrial genome of *A. cephalotes* is not annotated, the data available shows a genome with similar size and containing a large number of IGS.

Although we do not know the function of this IGS in Hymenoptera, it is interesting to note that a range of studies have reported an accelerated rate of gene rearrangement in mitogenomes of Apocrita, when compared with non-apocritans [19], [20], [43], [54]. Together, these data might suggest an association between IGS and number of rearrangements. Further studies characterizing the mitochondrial genomes of additional Hymenoptera species is needed to better understand the role and evolution of these non-coding sequences and the possible association with gene rearrangements.

In Formicidae, the mitogenome of *A. laevigata* was found to be 2,549 and 3,180 bp longer than that of *P. punctatus* and of *S. invicta*, respectively (Table 1, Figure 2). This difference was due primarily to the presence of IGS rather than differences in gene length. It has been noted that the size of the IGS between *COI* and *COII* genes increases from lower to higher Attini ants, honey ants, and bees [76], [77], [46]. Thus, variation in the size of the IGS is recognized as an evolutionary marker of social insects. Our data suggest that determination of the IGS position on the mitochondrial genome of Attini ants also may be valuable for phylogenetic studies. Because the IGS is highly variable [78] and informative for studies at subspecies level [79], it may be useful for distinguishing sibling species of Attini ants.

## Conclusions

We observed exponential growth in the number of published articles using NGS in the previous few years [80], [81], resulting in the availability of abundant NGS transcriptomic data containing valuable information regarding mitochondrial genes. As demonstrated in the present study, this information is important for initiating the assembly of whole genome sequences. Consequently, these data should be explored to generate more mitogenomes for different species, thus contributing to a better understanding of the phylogenetic relationships and evolutionary history of many groups of organisms.

Ants are a promising group for the application of this mitochondrial genome sequencing strategy, if we consider that *A. laevigata* mtDNA was only the fifth mitogenome annotated within over 12,000 described species with a dominant ecological role [11]. The mitochondrial genome of *A. laevigata* is the first one sequenced and annotated for the Attini tribe and can provide basic data for studies investigating population history, molecular systematics, and phylogeography, and also contribute to a better understanding of the mitochondrial rearrangements that occurred during Hymenoptera evolution.

## Supporting Information

Figure S1 **Primers used to amplify** ***A. laevigata*** **mitogenome.** Green: primers designed in this study; blue: primers obtained from the literature.
(TIF)

Figure S2 **Bayesian tree for all codon position and rRNA genes.** Posterior probabilities are shown at each node.
(TIF)

Table S1 Primers and annealing temperatures (Ta) for the *Atta laevigata* mitochondrial regions amplified.
(DOCX)
