# Supplementary figures and images for: The Mitochondrial Genome of the Leaf-Cutter Ant Atta laevigata: A Mitogenome with a Large Number of Intergenic Spacers
Source: PLoS One. 2014 May 14;9(5):e97117. doi: 10.1371/journal.pone.0097117 (PMC4020775; doi:10.1371/journal.pone.0097117)

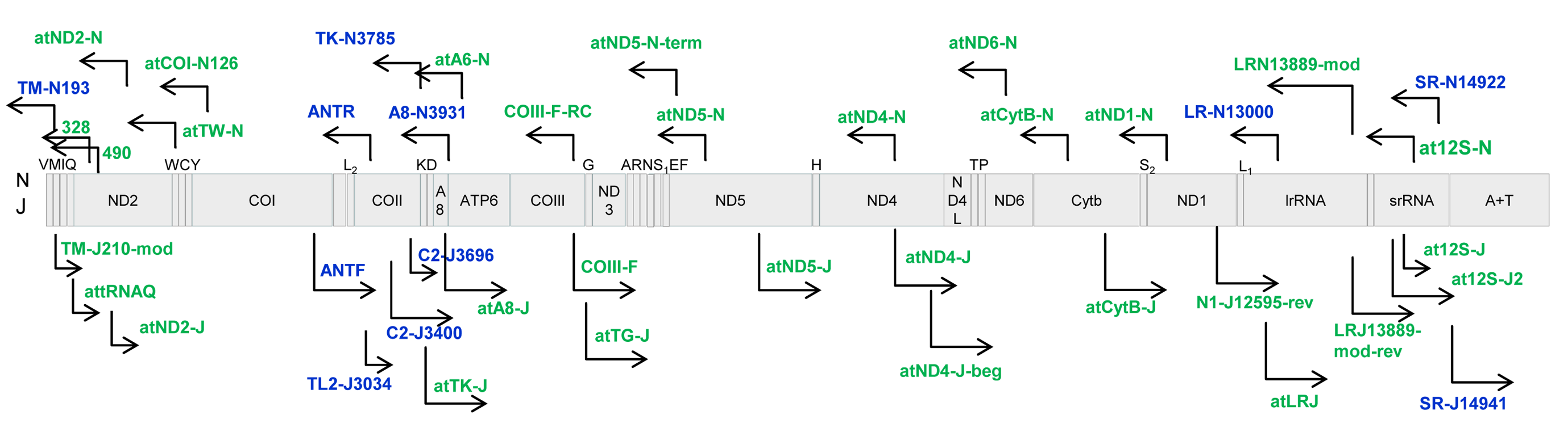

Supplement: Figure S1 — Primers used to amplify A. laevigata mitogenome. Green: primers designed in this study; blue: primers obtained from the literature. (TIF) [file pone.0097117.s001.tif]

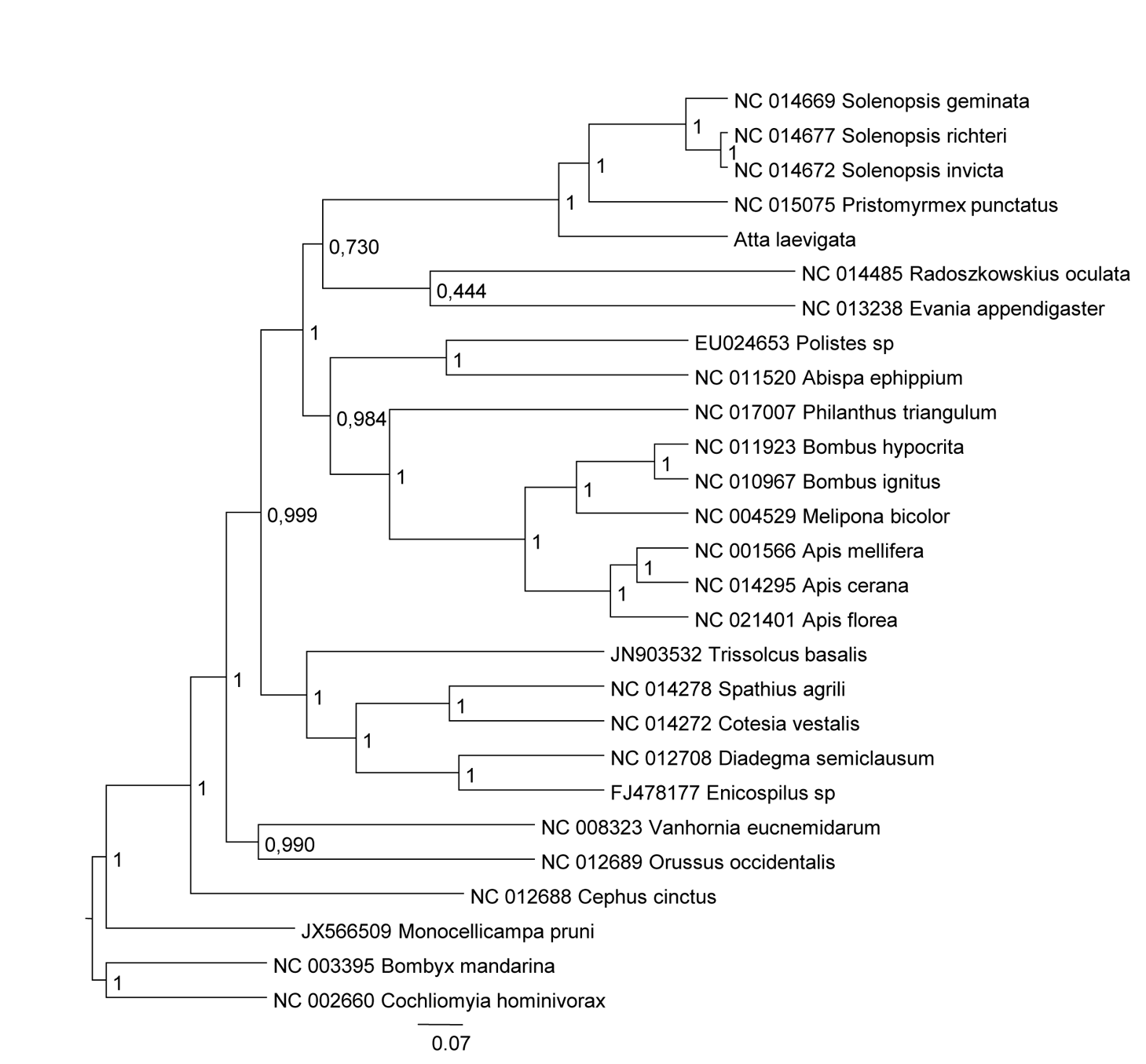

Supplement: Figure S2 — Bayesian tree for all codon position and rRNA genes. Posterior probabilities are shown at each node. (TIF) [file pone.0097117.s002.tif]
