# Supplementary material for: The Mitochondrial Genome of the Leaf-Cutter Ant Atta laevigata: A Mitogenome with a Large Number of Intergenic Spacers
Source: PLoS One. 2014 May 14;9(5):e97117. doi: 10.1371/journal.pone.0097117 (PMC4020775; doi:10.1371/journal.pone.0097117)
Supplement: Table S1 — Primers and annealing temperatures (Ta) for the Atta laevigata mitochondrial regions amplified. (DOCX) [file pone.0097117.s003.docx]

**Table S1.** Primers and annealing temperatures (Ta) for the *Atta laevigata* mitochondrial regions amplified.

| **Primer Name** | **Primer Sequence (5’-3’)** | **Ta (°C)** | **Amplified Region** | **Source** |
| --- | --- | --- | --- | --- |
| ANTF  ANTR | ATTCATTCTTATCTTGAAATATTATTTC  TTCATAAGTTCAGTATCATTGGTG | 47 | *COI_COII* | MARTINS et al., 2007^a^  MARTINS et al., 2007^a^ |
| C2-J3696  A8-N3931 | GAAATTTGTGGAGCAAATCATAG  AATTGGTGCTATTTGAGG | 50 | *COII_ATP8* | SIMON et al., 1994^b^  This study |
| TL2-J3034  TK-N3785 | AATATGGCAGATTAGTGCA  GTTTAAGAGACCAGTACTTG | 50 | *COII_tRNAK* | SIMON et al., 1994^b^  SIMON et al., 1994^b^ |
| SR-J14941  TM-N193 | AGCCAAAATAAAACTTTA  TGGGGTATGAACCCAGTAGC | 45 | Control region | OLIVEIRA et al., 2006^c^  SIMON et al., 1994^b^ |
| C2-J3400  COIIIFRC | ATTGGACATCAATGATATTGA  TAACATGAATTCCGTGGAATCC | 50 | *COII_ATP8_ATP6_COIII* | SIMON et al., 1994^b^  This study |
| C2-J3696  COIIIFRC | GAAATTTGTGGAGCAAATCATAG  TAACATGAATTCCGTGGAATCC | 50 | *COII_ATP8_ATP6_COIII* | SIMON et al., 1994^b^  This study |
| COIIIF  atND5N | GGATTCCACGGAATTCATGTTA  TTAACGGTGTCATATTCCTTACG | 50 | *COIII_NADH3_NADH5* | This study  This study |
| atND4J  AtCytBN | CAGGAGCCTCAACATGAGC  CCAAGTAATGAGCCAAAATTTG | 50 | *NADH4_NADH4L_NADH6_Cytb* | This study  This study |
| AtCytBJ  atND1N | CCTAATAAACTTGGGGGAGTAAT  GATGAGTTAATGTATTTATGTTGA | 50 | *Cytb_NADH1* | This study  This study |
| N1J12595-rev  LRN13000 | CGTTCTAATAAAGTTAAAAATGCTAC  TTACCTTAGGGATAACAGCGTAA | 50 | *NADH1_lrRNA* | This study  SIMON et al., 1994^b^ |
| N1J12595-rev  LRN13889-mod | CGTTCTAATAAAGTTAAAAATGCTAC  TGTACCTTTTGTATCAGGGTT | 50 | *NADH1_lrRNA* | This study  This study |
| LRJ13889-mod  at12SN | AACCCTGATACAAAAGGTACA  AAACTAGGATTAGATACCCTA | 50 | *lrRNA_srRNA* | This study  This study |
| atLRJ  at12SN | GATTTTAAAAGTCGAACAGAC AAACTAGGATTAGATACCCTA | 50 | *lrRNA_srRNA* | This study  This study |
| atND5J  atND4N | GAGACAGGAGTTGGAGCTGCTA  AAAGCTCATGTTGAGGCTCC | 45-53 | *NADH5_NADH4* | This study  This study |
| atND2J  atCOIN126 | TCTTCTATTAATCAATCTAGATG  ACATGATCTTAATTCTAATCG | 47 | *NADH2_COI* | This study  This study |
| atTKJ  atA6N | CATTAAATGACTGAAAAAGTATTG  GGATCAAAAATAGAAAATAAATTTATTATC | 47 | *tRNAK_ATP8_ATP6* | This study  This study |
| atA8J  atA6N | ATACCTCAAATAATACC  GGATCAAAAATAGAAAATAAATTTATTATC | 47 | *ATP8_ATP6* | This study  This study |
| atTGJ  atND5N-term | GTATAAATATTACATTTAATTTCC  AGATTATATAGAGTAGTGTATAAACC | 47 | *tRNAG_NADH3_NADH5* | This study  This study |
| atND4J-beg  atND6N | GAATAATAATTACACCCTAAAC  GAGTATATATAATTTTCTGATC | 47 | *NADH4_NADH4L_NADH6* | This study  This study |
| SR-J14941  atND2N | AGCCAAAATAAAACTTTA  TGAAAAAGGAGGTATGCCTG | 52 | Control region_*NADH2* | OLIVEIRA et al., 2006^c^  This study |
| at12SJ  TM-N193 | TGGATTATCATTTATAAGACAAATTCCTC  TGGGGTATGAACCCAGTAGC | 52 | *srRNA*_Control region_*tRNAM* | This study  SIMON et al., 1994^b^ |
| TMJ210-mod  atND2N | AGGGTATGAACCTAGTAGCTT  TGAAAAAGGAGGTATGCCTG | 50 | *tRNAM_NADH2* | This study  This study |
| at12SJ  atTWN | TGGATTATCATTTATAAGACAAATTCCTC  GGTTAATAGTTTAAATTTAAC | 47 | *srRNA*_Control region_*tRNAW* | This study  This study |
| at12SJ2  N490 | TTATGAAAATGACGGGCAATTTGTAC  GACTTAAAAAAGGTGGAATAATTGAGC | 52-58 | *srRNA*_Control region_*NADH2* | This study  This study |
| attRNAQ  N328 | GAGCATAAAAATTTTTGAAGTTTTTAG  ATCAAAAGTGGAAAGGAGGAATAC | 50 | *tRNAQ_NADH2* | This study  This study |

^a^ Martins Jr J, Solomon SE, Mikheyev AS, Mueller UG, Bacci M (2007)Nuclear mitochondrial-like sequences in ants: evidence from Atta cephalotes (Formicidae: Attini). Insect Molecular Biology 16: 777-784.

^b^ Simon C, Frati F, Beckenbach A, Crespi B, Liu H, Flook, P (1994) Evolution, weighting and phylogenetic utility of mitochondrial gene sequences and compilation of conserved polymerase chain reaction primers. Annals of the Entomological Society of America 87: 651-701.

^c^ Oliveira MT, Rosa AC, Azeredo-Espin AML, Lessinger AC (2006) Improving access to the control region and tRNA gene clusters of Dipteran mitochondrial DNA. Journal of Medical Entomology 43: 636-639.
